# Enhancement of COD Removal from Oilfield Produced Wastewater by Combination of Advanced Oxidation, Adsorption and Ultrafiltration

**DOI:** 10.3390/ijerph16173223

**Published:** 2019-09-03

**Authors:** Xiaodong Dai, Jian Fang, Lei Li, Yan Dong, Jianhua Zhang

**Affiliations:** 1 Shengli College China University of Petroleum, Dongying 257061, China (X.D.) (L.L.) (Y.D.); 2 China National Offshore Oil Corporation Group, Tianjin Chemical Research and Design Institute Corporation, Tianjin 300100, China; 3 Institute for Sustainable Industries & Liveable Cities, Victoria University, Melbourne, VIC 8001, Australia

**Keywords:** oilfield produced wastewater, Fenton oxidation, activated carbon adsorption, ultrafiltration membrane

## Abstract

The wastewater produced from the oilfield is chemically corrosive due to high salinity in combination with high temperatures. It is also rich in contaminants, such as oil, polyacrylamide, emulsions, suspended solid, etc. The density difference between the oil and water in the wastewater is low, which makes separation via gravity difficult. In this study, a combined pilot treatment is studied, which includes Fenton oxidation, settlement, activated carbon adsorption, and ultrafiltration (UF). The operational conditions of Fenton oxidation are optimized based on alleviating the fouling of the UF membrane. When the Fenton oxidation was operated at the molar ratio of H_2_O_2_ to FeSO_4_ 3:1 and pH 2.2–2.5, the UF membrane could operate continuously for 20 h without cleaning. The membrane was fouled by the organics (oil/grease) and polymer, which can be effectively removed by composite cleaning reagent consisting of 0.1% NaOH and 0.1% sodium dodecylbenzenesulfonate (SDBS). With the UF treatment, the chemical oxygen demand (COD) of the effluent was less than 50 mg/L, which could meet the upgraded standard.

## 1. Introduction

The oil exploitation industry is the foundation of almost all modern economic sectors. However, its processes also generate a large volume of liquid waste [1]. Oilfield wastewater or produced water contains various organic and inorganic components. Historically, the produced water was discarded in vast evaporation ponds, which would cause both ecological and social issues [2].

The wastewater produced from the oilfield is chemically corrosive due to high salinity in combination with high temperatures. It is also rich in oil emulsion, grease, suspended solids, bacterial content, and polymeric content, which could cause serious membrane fouling [3]. The low oil–water density difference and existing of surfactants make the separation of oil from the water difficult [3]. Since large volumes of the produced water are being generated in many countries with oilfields, there are increasingly focused efforts to find efficient and cost-effective treatment methods to remove pollutants. Reuse and recycling of the produced water include underground injection to increase oil production, use for irrigation, livestock or wildlife watering and habitats, and various industrial uses (e.g., dust control, vehicle washing, power plant makeup water, and fire control) [4]. Therefore, intensive treatment is required to meet regulations in the wastewater management or for wastewater recycling [5,6]. 

The main aim in treatment of oilfield produced water is to remove hydrocarbon components in terms of the chemical oxygen demand (COD) [7]. Oil content of the produced water can be reduced through various physical, chemical, and biological methods. In offshore oil extraction facilities, the compact physical and chemical treatment technologies are preferred, but they are limited due to the high capital cost of physical methods and high treatment cost of hazardous sludge generated from the chemical treatment [1]. Biological treatment is a cost-effective method for removing dissolved and suspended compounds from onshore oilfield wastewater. Several conventional methods have been used to treat the produced water for decades, which include flotation, coagulation, and biological treatment [2]. Both flotation and coagulation are physicochemical methods, and biological treatments utilize anaerobic and aerobic methods to reduced COD and biological oxygen demand. However, current methods cannot remove minute suspended oil and/or hazardous dissolved organic and inorganic components. Therefore, the effluent from those treatments hardly meets the most recent stringent standards [5,6], in which it is required that the COD must be less than 50 mg/L [7]. 

To achieve high COD removal efficiency, membrane filtration has been proposed to improve the effluent quality [8,9]. Zhang et al. [10] tried microfiltration technology in combination with coagulation to treat the produced water. Çakmakce et al. [11] tested the desalination of the produced water by the combination of dissolved air floatation, acid cracking, coagulation with lime and precipitation, cartridge filters, microfiltration and ultrafiltration (UF), and nanofiltration and reverse osmosis. Alkhudhiri et al. [12] tested air gap membrane distillation in treatment of the produced water. All those methods could achieve the targeted COD of less than 50 mg/L. However, in those methods, the upgrade of the existing treatment plant—which was still working and just could not treat the wastewater to the quality that met the new standard—has not been studied. 

Therefore, in this study, the combination of an existing conventional produced water plant with UF is proposed. The conventional produced water treatment plant with a maximum capacity of 480 m^3^/day was used as the pretreatment for UF process to reduce UF operation cost, which includes the Fenton oxidation, settlement, intermediate tank, and activated carbon units. The operation conditions of the pretreatment were optimized to lower the operation cost and improve the UF performance. The capability of the UF membrane in COD removal and cleaning strategy of the fouled membrane were also studied.

## 2. Experimental

### 2.1. Pretreatment

**Figure 1:**
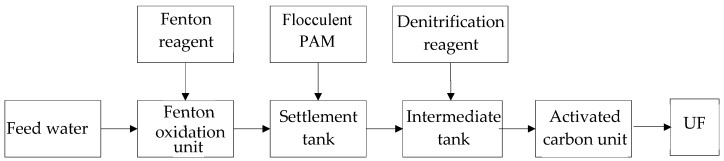
Treatment process. PAM: polyacrylamide; UF: ultrafiltration.

The maximum capacity of the pilot plant is about 480 m^3^/d, and the process schematic is shown in Figure 1, which was combination of an old conventional produced water treatment plant with UF. In the conventional pilot plant, the wastewater was fed into the Fenton oxidation unit, where part of the organic pollutants is oxidized and degraded by Fenton reagent, which will reduce the follow adsorption burden of the activated carbon and lower the fouling potential of the UF membrane. The flocculent (polyacrylamide (PAM)) is dosed into the settlement tanks equipped with inclined plates (board spacing of 100 mm), contacted with the effluent from the Fenton oxidation and settled down, through which the suspended solid and partial dissolved organic will be removed. The supernatant from the settlement tank enters the intermediate tank. Due to the incomplete oxidation in Fenton oxidation, NH_3_-N still can be detected in the intermediate tank and is further removed by adding a denitrification reagent (BH-TN07, Tianjin Research Institute for Water Transport Engineering, M.O.T, Tianjin, China), which will reduce the adsorption burden of the activated carbon. The denitrified effluent from the intermediate tank flows into the activated carbon unit for the adsorption procedure to further remove the dissolved organics and by-produce from the Fenton oxidation, which will reduce the fouling potential to the UF membrane. 

The operation process is controlled by a distributed control system (DCS). The pH in the Fenton oxidation unit is automatically adjusted by sulfuric acid addition. The sludge in the settlement tank is discharged based on the COD level in the effluent.

The components in the wastewater such as the surfactants, suspended solids, heavy metals, etc. are not analyzed in our study, because those chemicals are not regulated in the oilfield operation. Furthermore, during the testing period of about six months, no issue had been observed related to those components, although they have been widely studied and considered as factors that could potentially affect the performance of the Fenton oxidation, activated carbon, and UF. The water quality requirements for influent and effluent of the pretreatment are listed in Table 1. The COD is analyzed by the dichromate method (GB 11914-89), in which K_2_Cr_2_O_7_ is used to oxidize the COD in the analyzed water, and the NH_3_-N content is tested by an online ammonia analyzer.

#### 2.1.1. Fenton Advanced Oxidation Unit

The Fenton oxidation reactor consists of 3 reaction tanks (ϕ2400 × 3500 mm), which are made from Q235 carbon steel with internal phenolic resin coating and external polyurethane coatings for corrosion protection. A horizontal centrifugal pump able to deliver a flowrate of 50 m^3^/h is used to lift outlet flow, which is controlled by a static pressure level sensor at the following neutralization stage of the settlement tank.

#### 2.1.2. Settlement Unit and Intermediate Tank Unit

The settlement unit is consisted of a group of coagulation/flocculation tanks and inclined plate settlement tanks. The sizes of the reaction coagulation/flocculation and settlement tanks are 2700 × 2857 × 4510 mm and 6000 × 6000 × 4510 mm respectively. The effluent from Fenton oxidation unit flows into coagulation/flocculation tanks to separate the flocs. The supernatant from the coagulation/flocculation tanks is introduced into the inclined plate settlement tanks at a flowrate of 0.82 m^3^/m^2^·h, where its pH is adjusted to 9.0–9.5 to facilitate the iron precipitation and the following ammonia nitrogen removal [13,14]. 

The effluent from the settlement unit flows into intermediate tanks (3300 × 3300 × 2000 mm), where the denitrification reagent is added into the effluent from the settlement unit to reduce the ammonia nitrogen content. 

#### 2.1.3. Activated Carbon Adsorption Unit 

There are 6 sets of activated carbon adsorption tanks operating in parallel, which receive effluent from the intermediate tank unit. The size of the tank is ϕ2600 × 2300 mm with the phenolic resin internal coating and polyurethane external coating for corrosion protection. 

### 2.2. UF Membrane Separation Unit

The pretreatment process was designed to meet the previous requirements for wastewater discharge, but it could not meet the more stringent standard currently. Therefore, an UF membrane unit was added following the activated carbon unit, and the investigation was conducted to optimize the pretreatment process under the new operation conditions [15,16].

The UF membrane unit includes 24 polyethersulfone hollow fiber membrane modules (effective membrane area of each element = 40 m^2^). The maximum operating pressure and temperatures of the modules are 0.20 MPa and 50 °C respectively. The molecular weight cut off (MWCO) of the membrane is 60,000 Dalton. The UF unit was challenged by the feed without the pretreatment, and then challenged by the effluent post the whole pretreatment process. 

## 3. Investigation of Pilot Test Parameters

### 3.1. Fenton Oxidation

#### 3.1.1. H_2_O_2_ and FeSO_4_ Addition

The efficiency and operation cost of Fenton oxidation procedure are directly related to the dosing of H_2_O_2_, which is determined by COD in the feed water. In these tests, the H_2_O_2_ d was maintained at 300 mg/L. The Fenton reaction is shown in Equations (1)–(3) [17]:

(1) $$H_{2}O_{2}+{\mathrm{Fe}}^{2+}={\mathrm{Fe}}^{3+}+{\mathrm{OH}}^{-}+\mathrm{HO}\cdot$$

(2) $$\mathrm{HO}\cdot +{\mathrm{Fe}}^{2+}={\mathrm{Fe}}^{3+}+{\mathrm{OH}}^{-}$$

(3) $$H_{2}O_{2}+{\mathrm{Fe}}^{3+}=\mathrm{Fe}-{\mathrm{OOH}}^{2+}+H^{+}$$

**Figure 2:**
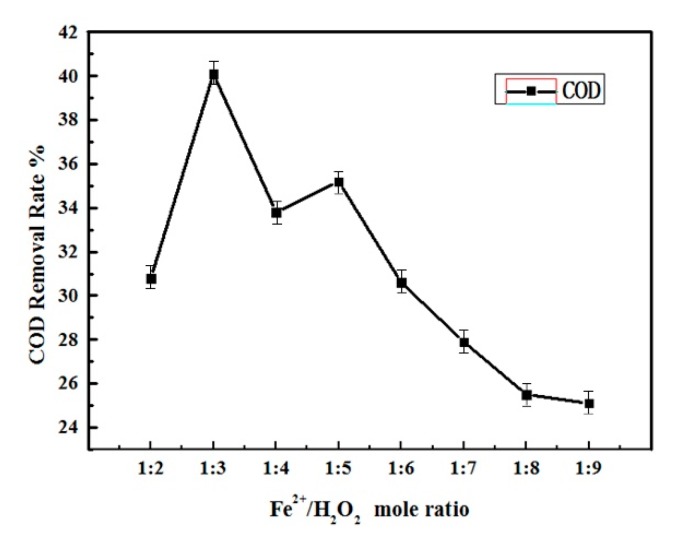
Chemical Oxygen Demand (COD) removal with Fe^2+^/H_2_O_2_ mole ratio (pH = 1.9–2.2) (error = ± 5%).

In the reaction, the Fe^2+^ acts as a catalyst. Therefore, its concentration will affect the formation of the OH free radicals and the performance of the Fenton oxidation. To find the optimal dosing of Fe^2+^, the COD removal efficiency based on the different mole ratios of Fe^2+^/H_2_O_2_ was studied, which is shown in Figure 2. It can be seen that with the increment of the molar ratio of Fe^2+^/H_2_O_2_, the COD removal rate increased and reached the maximum removal rate of 40% when the molar ratio of Fe^2+^/H_2_O_2_ is 1:3. The COD removal efficiency reduced again as the Fe^2+^ concentration increased further.

Since the H_2_O_2_ dosing into the wastewater maintained the same in the tests, the higher Fe^2+^ concentration in the solution would increase the effective production of hydroxyl radicals [17]. However, it also can be found from Equation (2) that the Fe^2+^ could also consume the newly formed OH radicals, since it is also an active reducing reagent [17]. Therefore, there is a trade-off of the Fe^2+^ addition to achieve the maximum COD removal efficiency.

The Fenton oxidation will produce some by-products, which is more toxic sometimes [18]. However, the activated carbon adsorption could remove those by-products.

#### 3.1.2. pH Adjustment

**Figure 3:**
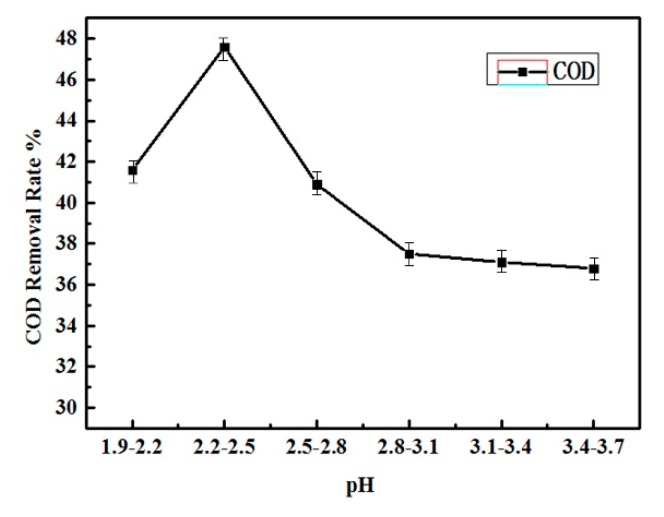
COD removal with pH value in Fenton Unit (Fe^2+^/H_2_O_2_ molar ratio = 1:3) (error = ±5%).

The pH of the feed will also affect Fe^2+^ and Fe^3+^ equilibrium and the Fenton oxidation result [19,20]. The influence of pH on COD removal is shown in Figure 3, in which the pH was monitored by an on-line pH meter and adjusted by H_2_SO_4_ addition. It can be found from Figure 3 that the feed pH is in a range rather than a specific value due to the Fenton oxidation producing OH and pH adjustment lagging [17]. The COD removal rate increases as the pH becomes lower, and achieves the maximum value of 48% when the pH is in the range of 2.2–2.5, as shown in Figure 3. When the pH reduced further, the COD removal rate declined again.

As shown in Equations (1) to (3), the H_2_O_2_ decomposition will produce OH^−^ or consume H^+^ Therefore, the lower pH will reduce the decomposition rate of H_2_O_2_ and prolonged its reactivity [17]. Furthermore, the lower pH in an acidic range could prevent the dissolved iron from precipitation, which maintains the Fe^2+^ catalyst concentration in the solution. However, if the pH is too low, as it can be seen from Equation (3), the high H^+^ concentration will suppress the transformation from Fe^3+^ to Fe^2+^, which will substantially lower the Fe^2+^ concentration as catalyst. Therefore, the optimal pH is in the range of 2.2–2.5.

### 3.2. Settlement and Intermediated Units

The settlement unit receives effluent from Fenton oxidation unit. The pH of the effluent from the Fenton oxidation unit was adjusted to 9.0–9.5, and 30 mg/L PAM was dosed as the flocculent into the settlement unit to enhance the settlement. The effluent from the settlement unit was introduced into the intermediate tank, where 500 mg/L denitrification agent was added to control NH_3_-N lower than 8 mg/L by forming insoluble Struvite salt [13,14]:

(4) $${\mathrm{Mg}}^{2+}+{\mathrm{NH}}_{4}^{+}+H_{n}{\mathrm{PO}}_{4}^{n-3}+6H_{2}O↔{\mathrm{MgNH}}_{4}{\mathrm{PO}}_{4}\cdot 6H_{2}O+{\mathrm{nH}}^{+}$$

### 3.3. Activated Carbon Adsorption 

The coal-based activated carbon U-4X was used as the adsorbents. The COD of effluent was controlled in the range of 150–170 mg/L after adsorption, which was designed to meet the old discharge standard. The activated carbon will be replaced or regenerated after 1000 m^3^ water have been treated. The treatment capability of the activated carbon is directly related to the residence time (plant load) [21]. Short residence time or high plant load would result in the quality of effluent from the activated carbon process not meeting the old regulation. 

### 3.4. UF Membrane Challenge Tests

#### 3.4.1. Influence of Operating Pressure on Membrane Flux and Water Quality

**Figure 4:**
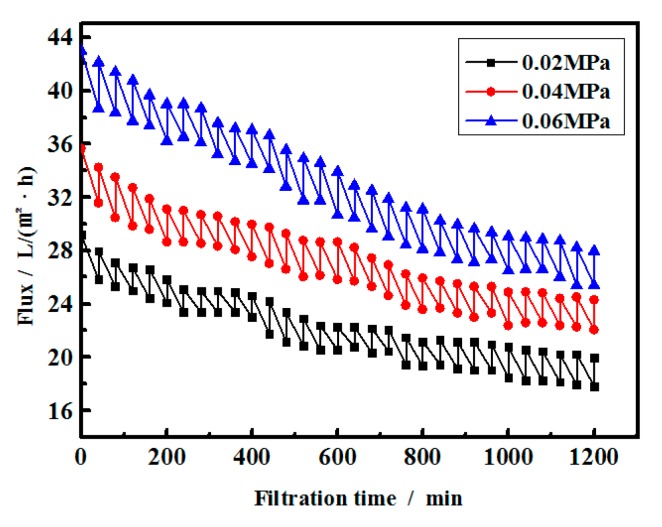
Effect of operating pressure on ultrafiltration (UF) flux.

The transmembrane pressure will directly affect the permeate flux of UF. Based on the influent quality, the backwashing cycle and backwashing time were set at 39 min and 40 s respectively. The membrane fluxes were compared at transmembrane pressure of 0.02 MPa, 0.04 MPa and 0.06 MPa as shown in Figure 4. It can be observed that the initial flux of the membrane is higher at higher transmembrane pressure, and all fluxes gradually decreased with operating time. However, membrane flux decrease percentages are different under different operation pressures for the same operating time of 1200 min, which were 37.68%, 37.94%, and 40.96%, respectively, at the transmembrane pressures of 0.02 MPa, 0.04 MPa, and 0.06 MPa. The greater and faster flux decline under high transmembrane pressure is due to the formation of the thicker and denser fouling cake under higher operation pressure [22]. Furthermore, oil droplets larger than the pore size may permeate the membrane if the transmembrane pressure is great enough [23], and cause COD increase in the permeate. Therefore, the selection of operation pressure should also consider reducing the oil droplet penetrating risk, besides high and stable fluxes. From Figure 4, it can be found the UF operating pressure of 0.04 MPa could maintain a relative high and stable flux and no oil penetration was detected based on Table 2. Thus, the transmembrane pressure would be the optimal operation pressure when the studied feed is used. 

The water quality after UF treatment is shown in Table 2. It can be seen that after 20 h, UF filtrate still met the design requirements under all the tested conditions. Attributing to the high COD of the poly-bearing wastewater and the high suspended solid content in the water, it can be found from Table 2 that the 15-min Silt Density Index (SDI_15_) varied with both time and pressure. It also can be found that as the pressure becomes greater, the SDI_15_ in UF filtrate increases, which suggests some particulates were forced through the membrane under high pressure. 

It can be found under high operation pressure (0.04 and 0.06 MPa), the suspended solid and oil content reduced with time. It is due to irreversible fouling, which substantially reduces the membrane pore size [24,25]. As results, the UF membrane showed an improved performance in the suspended solid and oil content removal.

#### 3.4.2. Chemical Cleaning for Membrane 

In this study, the cleaning efficiency of five different chemical cleaning reagents with 0.1 wt% concentration were investigated and compared at 25 °C. The cleaning efficiencies were compared based on the pure water flux recovery factor (*r*):

(5) $$r=\frac{J_{Q}}{J_{O}}\times 100\%$$

where *J_Q_* is the pure water permeate flux of the membrane after cleaning, L/(m^2^·h); *J_O_* is the initial pure water permeate flux of the membrane, L/(m^2^·h)

**Figure 5:**
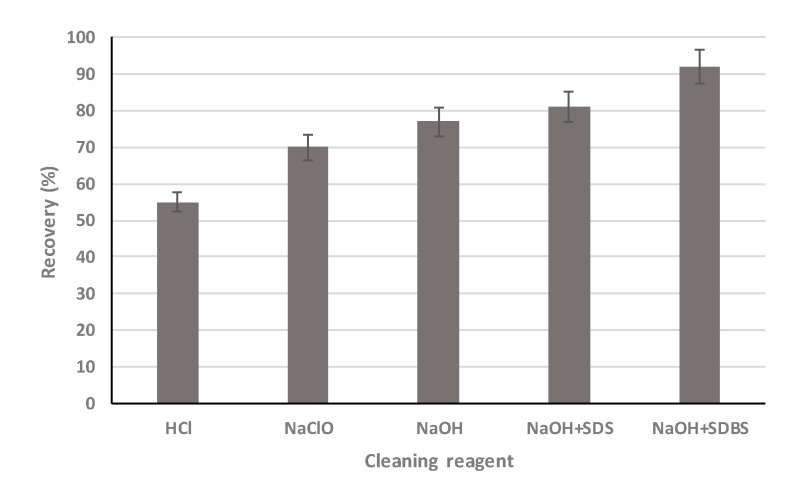
Efficiency comparison of different chemical cleaning reagents (error = ±5%).

In Figure 5, it is observed that the 0.1 wt% HCl cleaning led to the lowest flux recovery of 55%, indicating that the inorganic scale is not the main foulent for the UF membrane. Cleaning with 0.1% NaOH and 0.1% NaClO recovered the membrane flux to 77% and 70%, respectively, which were better than that of HCl cleaning, because the organics are majorly foulents during the treatment [8,26,27]. Furthermore, it can be seen that the NaClO cleaning recovered less flux than that of NaOH cleaning, which means the oxidation affects the foulents less than that of the alkalinity after the Fenton oxidation. However, flux recovery by NaOH cleaning was still less than 80%, which indicates that membrane fouling was not just caused by petroleum substances [28].

Therefore, two composite cleaning reagents containing two different types of surfactants were tested. About 81% flux was recovered after 0.1% SDS and 0.1% NaOH cleaning, which is just a little better than 0.1% NaOH cleaning. Since SDS is highly effective in any task requiring the removal of oily stains and residues [29], some foulents seem not from the oil. It can be found that the wastewater also contains polymer, which has a strong binding force with the membrane surface and would cause the membrane flux decline. Since sodium dodecylbenzenesulfonate (SDBS) is a surfactant that can effectively remove granular fouling [30], it was tested in the cleaning process. It can be found from Figure 5 that with 0.1% NaOH and 0.1% SDBS cleaning, more than 90% flux recovery was achieved. Therefore, it is demonstrated that the organics (oil/grease) and polymer fouling in the produced water are the main foulents causing the UF flux decline.

### 3.5. Treatment Capacity Challenge for the Combined Process

The analytical data from the combined process are shown in Table 3, where the pretreated water was used as the UF feed. As the inflow rate increasing, it can be seen that the COD and turbidity of the post-UF water increased. When the inflow rates are the 15 and 20 m^3^/h, the CODs of the effluent from the activated carbon adsorption treatment (Pre-UF water) are 186 and 201 mg/L, which are out of the required range of 150–170 mg/L. However, even at the highest inflow rate of 20 m^3^/h (membrane flux = 21 L/m^2^·h), the COD of the UF filtrate (Post-UF) was still less than the designed value of 50 mg/L. Similar results are also reported by Bilstad and Espedal [28,31] that hydrocarbon content decreases from 50 mg/L to 2 mg/L, post the UF treatment to the produced water for North Sea oilfield. Therefore, by the combining the pretreatment with UF, the process guarantees the quality of the product water meet the updated standard.

### 3.6. Cost Analysis

The treatment cost of the produced water varies dramatically, according to the calculation method and water quality [32]. Ersahin et al. estimated the operation cost (including reverse osmosis) about 0.88 US$/m^3^ [33]. However, the comprehensive cost of the studied pretreatment system is about 30 CNY/m^3^ waste water (about 4–5 US$/m^3^), which includes 30% of operation cost (labor, electricity and chemical), and 70% of maintenance cost (general annual maintenance, service team, sludge removal, and activated carbon regeneration or replacement). With the UF Extra treatment, 5–8 CNY/m^3^ (about 1 US$/m^3^) is added upon the pretreatment cost, which increases the total cost less than 20%. Therefore, the total cost for the treatment combination is about 5–6 US$/m^3^. Furthermore, the small footprint of the UF membrane assembly [19] makes it able to fit into the spare space of the pretreatment process. Compared to directly dealing with the raw oilfield produced water, the chemical cleaning frequency for the membrane with the pretreatment is also reduced, which lowers the chemical consumption cost.

## 4. Conclusions

An upgrade of an existing conventional plant including Fenton oxidation, settlement, and activated carbon adsorption is studied. With the combination of UF into the existing plant, the COD reduced from 150–170 mg/L to below 50 mg/L, which complies with the stringent new regulations.

The UF membrane was challenged with the feed prior to the pretreatment initially to obtain a conservative operation condition. It was found that after operating for 1200 min, 92% flux of the UF membrane could be recovered by cleaning with 0.1% sodium hydroxide and 0.1% SDBS mixture.

The Fenton oxidation performance could be enhanced by increasing the Fe^2+^ concentration or reducing the pH, with the same amount of H_2_O_2_ dosing. However, the very low pH (1.9–2.2) and high Fe^2+^ concentration (Fe^2+^/H_2_O_2_ > 1/3) would also suppress the COD reduction in the studied system.

In the pretreatment and UF combined tests, at the maximum pilot plant capacity, the UF filtrate still can meet the required standard (COD < 50 mg/L) under the conditions of

- Fenton advanced oxidation reaction: molar ratio of H_2_O_2_ to FeSO_4_ is 3:1, pH 2.2–2.5,
- Settlement tank: pH 9.0–9.5, PAM dosing: 30 mg/L, and
- UF membrane unit: transmembrane pressure 0.04 MPa

The polymer and organics (oil/grease) are the major foulents to the UF membrane, which can be effectively removed by using 0.1% NaOH and 0.1% SDBS as cleaning reagents.

With UF treatment, the COD of the treated water could meet the required discharge standard of 50 mg/L, even if the UF feed is out of the specification (150–170 mg/L).

With the addition of UF unit, the total cost increased about 20% to 5–6 US$/m^3^, in comparison with the conventional treatment.

## Figures and Tables

**Table 1:** Required influent and effluent parameters.

Parameters	pH	Chemical Oxygen Demand (COD) (mg/L)	Nitrogen (mg/L)
Influent	8.1	261	10.8
Effluent	6–9	≤170	≤8

**Table 2:** Analysis of UF filtrate.

Testing Item	Pressure (MPa)	Operating Time (min)		
		100	600	1200
Suspended solid (mg/L)	0.02	1.32	1.26	2.52
	0.04	2.16	2.94	2.56
	0.06	3.16	3.43	2.10
Oil content (mg/L)	0.02	0.00	0.02	3.86
	0.04	0.00	0.09	0.00
	0.06	0.02	0.02	0.00
SDI_15_ (15-min Silt Density Index)	0.02	1.24	2.58	3.76
	0.04	3.55	4.29	3.41
	0.06	3.90	4.68	5.76
Turbidity (Nephelometric Turbidity Unit)	0.02	0.02	0.08	0.05
	0.04	0.05	0.01	0.04
	0.06	0.03	0.06	0.04

**Table 3:** Analytical data of combined process.

Inflow (m^3^/h)	Fenton Oxidation Time (min)	Activated Carbon Adsorption Time (min)	COD (mg/L)		Turbidity (NTU)	
			Pre-UF	Post-UF	Pre-UF	Post-UF
5	320	480	139	13.0	138.8	9.09
10	160	240	145	20.6	154.2	9.72
15	106	160	186	25.1	180.2	11.37
20	80	120	201	26.7	186.3	14.06

Pre-UF: The CODs of the effluent from the activated carbon adsorption treatment; Post-UF: COD of the ultrafiltration filtrate.
